# Supplementary material for: Undergraduate occupational therapy students’ perceptions of their preparedness for splinting in hand rehabilitation: An exploratory study at the University of KwaZulu-Natal
Source: PLoS One. 2026 Feb 13;21(2):e0343017. doi: 10.1371/journal.pone.0343017 (PMC12904425; doi:10.1371/journal.pone.0343017)
Supplement: Attachment — Submitted filename: Review of PlosOne article 14052025. entitled.docx [file pone.0343017.s001.docx]

Review of article entitled: Undergraduate occupational therapy students’ perceptions of their preparedness for splinting in hand rehabilitation: an exploratory study at the University of KwaZulu-Natal- PONE-D-25-01929

General:

This worthwhile research identifies an area of occupational therapy (OT) undergraduate (UG) practical learning that is of importance but inadequately addressed in the institutional curriculum where the research was conducted. Reference is also made to the paucity of information in available literature on why students feel inadequately prepared for the use of splinting in clinical practice. Socio-economic barriers to the use of splinting are raised. The concern that poor splinting technique will deter graduates from specialising in hand rehabilitation is articulated. All these factors attest to the justification for the carrying out of this research.

The use of qualitative research methodology is appropriate with attention being paid to trustworthiness measures, ethical considerations and thematic analysis. However, some more detail related to aspects of the data collection needs to be given. The collected data in the form of quotations is of good quality and rich in useful information which suggests the open-ended questions were well designed and delivered.

The interpretation of results and conclusions offer suggestions and solutions to mitigate the curricular problems with the current practical teaching of splinting.

Abstract:

Well written, succinct and includes all the main points relevant to each section of the study.

Background:

Overall good organisation of information and relevant content.

Minor corrections

Page 5, Line 67: Is the use of the word “occupational” necessary?

Page 5, line 68: As the wording occupational therapy is used a lot perhaps consider introducing the abbreviation OT here as in occupational therapy (OT).

Page 5, lines 77-78: This point needs a citation.

Page 5, lines 79-80: This sentence is a little confusing. Perhaps consider using the word modality.as in:” which serves as a **modality that is** a crucial therapeutic….”

Page 5, line 83: There are instances in the manuscript where references and wording are repeated as here. Please check for similar problems elsewhere in the document and correct them.

Research question:

Minor corrections

Page 6, line 96: Suggest the addition of OT to students (to qualify who the students are) if using the abbreviation or occupational therapy students if writing in full.

This aligns with terminology to describe students in the Aim and objectives.

Aim:

Minor correction

Page 6, line 100: Could abbreviate occupational therapy to OT here.

Literature review:

Minor corrections

Can apply the abbreviation OT where appropriate.

Page 7, lines 113-114: this sentence raises the questions- How will healthcare access be enhanced? Which population’s well-being will be improved? Please clarify.

Page 7, lines 131-133; This sentence is confusing. Do occupational therapists move to the private sector due to lack of **resources** like splinting materials?

Page 8, line135: I would query the use of “apparent “here because the rest of the sentence suggests a definite shortage of occupational therapists.

Page 8, lines 138-140: “…the uneven distribution of occupational therapists is **due to** the limited number…” The addition of this wording helps with clarity. I would also suggest dividing the original long sentence into two starting the second one “ **This** makes it difficult….

Page 8, line148: human**s**

Page 8, line 151: Introduce the abbreviation multidisciplinary team (MDT) in this line as the abbreviation is used in line 153.

Page 9, line 162: I am interested to know how psychology is involved in restoring the function of the hand…

Page 9, line 183: Abbreviation UKZN already introduced.

Page 11, line 215: Abbreviate to MD.

Page 11, line 218: “emphasises**”**

Methodology:

What was the initial question used in the semi-structured interview? Who conducted the interviews? Were different researchers involved? Were meetings held during data collection to make sure all researchers were following the same questioning technique and adapting to changes in questioning brought about from insights from the data collection process? Were any incentives offered to participants such as free data or vouchers etc?

Was an inductive method used to analyse data?

Was verbatim transcription of interviews carried out timeously? Who did this? Was it done in Microsoft Word?

Who undertook the coding of the data? Was this done independently by researchers assigned to the task?

Minor corrections

Page 12, line 243: “**was** followed…. **were** adhered to**.”**

Page 12, line 257. Was approval for carrying out the research also obtained from the Head of the Occupational Therapy Department and the Dean of Student Affairs? This information could be added here or in the Ethical considerations section on Page 14.

Pages 12 and 13, lines 259-261: How and where was this data stored? Was the data anonymised? Did the information sheet give the rights of participants to confidentiality and the right to stop participation at any time **with no consequence**? Perhaps add to line 267-268.

Page 13, line 262: give reference for UKZN ethical policy.

Page 13, line 277; I would suggest removing wording “refer to title section for their credentials.”

Page 13, line 281: Perhaps add **anonymised** data to negate the chance of the reader thinking that there is any chance that identifiers would allow participants to be identified.

Results:

Rich data were collected and this allowed for pertinent and comprehensive quotations to be realised. This section is well laid out and quotations are appropriately used.

Page 16, Table 4.2; under Theme one, Subtheme 1.3, Theme three, Subtheme 3.2

I suggest that one of the quotes in the text under these Subthemes be moved from the text and used in the table.

Discussion

Although there is no specified word limit for this journal, I would suggest that perhaps in some instances fewer quotations could be used under the themes/subthemes. Some quotations basically say the same thing so the most comprehensive could be chosen. The use of a 3^rd^ year and a 4^th^ year quotation is good where possible to highlight differences in perceptions on a theme by students in different years of study.

Page 31, line 648: It is not preferred to begin a sentence with an abbreviation… in this case UKZN.

In general, the main points relating to undergraduate occupational therapy students’ perceptions of the preparedness for splinting in hand rehabilitation are dealt with and discussed in the discussion.

Sometimes in qualitative research “outliers” are identified in the data collection.

Perhaps consider adding a short paragraph on the dangers associated with inadequate splint making instruction.

For example: Could the lack of practice and competency in splint making and application lead to splints that are too “tight”? Could that lead to further problems for a patient? Or the fact that students recognised that the lack of adequate practice could lead to “endangering your client” and “it’s very dangerous to put a person in a splint they’re not prepared for.” Why? Does the available literature deal with this?

Conclusion

Concise and relevant to the study findings.

Limitations:

Well-articulated and identifies the four points of limitation clearly.

Recommendations:

These are very useful suggestions for addressing the problems identified in the study. I hope that at least some of these well thought out and useful ideas will be implemented.

Referencing:

Sequence of numbering of citations starts being chronologically inconsistent on Page 12 after reference 17. I’m not sure citation 18 is included in the manuscript. Please check.
