# Supplementary material for: Undergraduate occupational therapy students’ perceptions of their preparedness for splinting in hand rehabilitation: An exploratory study at the University of KwaZulu-Natal
Source: PLoS One. 2026 Feb 13;21(2):e0343017. doi: 10.1371/journal.pone.0343017 (PMC12904425; doi:10.1371/journal.pone.0343017)
Supplement: Attachment — Submitted filename: Responses to academic editor and reviewers.docx [file pone.0343017.s002.docx]

**Rebuttal letter - responses to academic editor and reviewer(s)**

- Please refer to the main manuscript (with no track changes) for accuracy.

| PLOS ONE comments | Authors’ comments  (insert your response under this column) |
| --- | --- |
| **Additional Editor Comments:** | Not applicable (N/A) |
| Dear authors,  Thank you for your submission to PLOS ONE. We appreciate the time and effort you have invested in this work. Following my detailed review and external reviewers' opinions, I have reached a decision that the manuscript requires major revisions before it can be reconsidered for publication. |  |
| While the topic addresses an important area, the manuscript in its current form requires significant enhancement in several critical aspects. |  |
| - The manuscript currently lacks a clear articulation of its original contribution. You are strongly advised to generate a compelling research gap, establish the significance of the study, and make a strong case for why this research is needed in your specific context. Explain how your work adds value beyond what is already well established in existing literature. | Consider the following:  "This study contributes original insight by documenting the lived experiences of UKZN occupational therapy students, highlighting institution-specific challenges and potential curriculum improvements in a resource-constrained setting."  "Unlike prior research, this study also uncovers nuanced differences in preparedness between third- and fourth-year students and presents concrete, student-driven solutions..."  Few studies have investigated this issue in-depth within the South African context, particularly at institution-specific levels such as UKZN."  "This study addresses this gap by exploring the specific perceptions of UKZN occupational therapy students..."  ...it contributes to the limited body of knowledge on how contextual factors influence student preparedness in splinting..." |
| - Please elaborate on why this study is relevant within your specific population or setting. Contextual justification is crucial to demonstrate the study’s relevance and applicability. | Refer to the last paragraph of the background and the last paragraph of the literature review.  “Despite national curriculum standards, the local challenges—such as resource constraints and variable fieldwork exposure—create unique training experiences..."  “Given the high burden of hand injuries in KZN and the uneven distribution of therapists across public and private sectors, this study is crucial in understanding how current training methods impact students’ competence and long-term workforce readiness.” |
| - The sampling strategy is unclear and requires further clarification. Describe how participants were selected and justify the use of the sampling method. Additionally, please explain how you ensured the trustworthiness and accuracy of self-reported data, especially considering potential biases. | Refer to population under methodology – “A purposive sampling strategy was used to select participants who had been exposed to splinting theory and practice, which is introduced in the 3^rd^ year of study. This method was chosen to ensure the inclusion of students with relevant knowledge and experience related to the research topic”  Corrections have been made to clarify the sampling strategy.  Refer to data collection, under methodology- Trustworthiness and accuracy of the data were ensured through several strategies grounded in credible qualitative research practices. Participation in the study was voluntary, and to promote anonymity and honest responses, participants were asked to refer to themselves as “Participant” during online Zoom interviews. All interviews were audio-recorded with participant consent and later transcribed verbatim to maintain the accuracy and integrity of the data. A consistent, pre-developed interview guide was used across all interviews to ensure uniformity in data collection. This guide was pilot tested on a group of occupational therapy students who had graduated the previous year.....’ |
| - The discussion does not meet the standards of effective academic debate. It should critically compare your findings with existing literature, reflect on contradictions and confirmations, explore theoretical and practical implications, and address limitations and directions for future research. | Refer to the revised sections: Discussion, Conclusion, Recommendations and impact on practice  The whole discussion had been revised. |
| - As currently presented, the conclusion is not well-supported by the findings, and no actionable implications for practice, policy, or research are clearly stated. Please revise to include concrete takeaways that readers can apply or build upon. |  |
| - The manuscript contains numerous awkward and unclear statements, along with grammatical and stylistic issues that hinder readability. We advise extensive English language editing to improve clarity, flow, and academic tone. Please ensure that your writing adheres to scholarly conventions throughout. | The entire document wise revised |
| Reviewer's Responses to Questions | |
| Reviewer #1: Thank you and congratulations for the interesting idea, but I must say that unfortunately the sample size is very small. Also, for such a decision, the study should be conducted simultaneously in several centers in different locations. | Under limitations, refer to bullet two and the last bullet. E.g.,:  “...Increasing the response rate can be achieved through follow-up reminders, simplifying data collection tools, and emphasising the value of participation. Expanding the sample size by including students from different year levels and even alumni can improve representation. Broader literature searches and collaboration with other institutions can provide more comprehensive and generalisable findings.” |
| Reviewer #2: This manuscript requires a significant amount of improvement in: | N/A |
| Follow the Consolidated criteria for reporting qualitative studies (COREQ) chick list | The entire methodology section was revised based on COREQ |
| **Introduction**  What is the novelty of the current study | In South Africa, OT undergraduate students, including those at the University of KwaZulu-Natal (UKZN), must complete at least 1,000 clinical or practical hours [6]. However, existing literature acknowledges students' lack of preparedness for splinting, yet few studies have investigated this issue in-depth within the South African context, particularly at institution-specific levels such as UKZN.  **The novelty of this study lies in its institution-specific focus, offering contextually grounded insights that can inform curriculum development and practical training strategies tailored to UKZN's particular environment.** |
| What is known about the topic? (Background) | Refer to background, paragraph two of the background, where hand rehabilitation as an area within occupational therapy is explained as well as splinting as part of hand rehabilitation. Also refer to literature review, as existing literature related to this topic is evident in the literature review. |
| What is not known? (The research problem) | Refer to paragraph three under background, this paragraph explains what had not been studied and purpose of this study, “However, existing literature broadly acknowledges students' lack of preparedness for splinting, yet few studies have investigated this issue in-depth within the South African context, particularly at institution-specific levels such as UKZN....” |
| Why the study was done? (Justification) | Splinting plays a critical role in hand rehabilitation, and occupational therapy practise. Understanding undergraduate occupational therapy student’s perceptions of their preparedness forb splinting can help identify educational gaps, improve clinical training, enhance confidence and ensure safe, effective patient care upon qualifying as an occupational therapist. |
| **Methods**  How the study was designed?  How the study was carried out?  How the data were analyzed?  What is the statistical design used in the data analysis | Refer to the entire methodology section that was revised based on COREQ |
| **Results**  Results need to provide answers to the questions raised/researchable problem | Research question: What are the perceptions of OT students on undergraduate splinting as a part of hand rehabilitation at the University of KwaZulu-Natal?  Answer: Themes one to for – refer to table 2 for an overview |
| Results need to follow ABC(accuracy, brevity, clarity)  Kindly frame it along the following elements of results  Kindly frame it along the following elements of results  Text to tell the story  Tables to summarize the evidence  Figures to highlight the main findings | These we covered as follows:   - The results section focuses on findings under the participants' (students') demographics and four emerging themes. - Demographics: provide an overview of who the participants were - Themes: Four themes and corresponding subthemes emerged in this research, summarised in Table 2 below. The four themes are student readiness and preparedness, challenges in splinting, comparison of readiness in 3rd year and 4th year, and suggested solutions to bridge the gaps. The themes are explored using verbatim quotes to authentically capture and represent the participants' perspectives. |
| **Discussion**  The discussion section needs to be described scientifically. Kindly frame it along the following lines:  • Main findings of the present study  • Comparison with other studies  • Implication and explanation of findings  • Strengths and limitations  • Conclusion, recommendation and future direction.  • Describe sources of potential bias and imprecision.  • Generalisability of the trial findings need to be put. | Refer to the revised sections: Discussion, Conclusion, Recommendations and impact on practice.  These are some supporting statements made throughout the manuscripts:   - It is mentioned as follows in the manuscript “Reflexivity was maintained throughout the research process to minimise bias” - The researchers relied on data from the participants as opposed to imposing their biases. |
| Reviewer #3: Your study addresses a critical and underexplored area in occupational therapy training—student readiness for splinting in hand rehabilitation. The context of KwaZulu-Natal and the broader implications for health equity add value. However, to align with PLOS ONE’s standards for qualitative research, the manuscript requires additional methodological clarity, a deeper engagement with global literature, and improvements in structure and presentation. | Refer to the revised:   - Method adhering COREQ - Literature review which now includes a section titled Kolb’s Experiential Learning Theory |
| Abstract: Be more specific in the abstract regarding the nature of student unpreparedness (e.g., lack of clinical opportunities, limited supervision). Add a sentence to highlight potential curriculum implications or policy relevance. | Revised conclusion. E.g., “These findings have implications for curriculum development and suggest the need for policy reforms prioritising clinical competency in undergraduate occupational therapy education” |
| **Introduction**  - Reduce redundancy (e.g., avoid repeating “splinting is a crucial technique” multiple times). | Corrections made |
| - Sharpen the focus on the specific knowledge gap your study addresses—how student confidence and competence in splinting is affected by current training models. | Refer to revised paragraph 3 in background focusing on the sentences : “Despite national curriculum standards, the local challenges, ~~—~~such as resource constraints and variable fieldwork exposure, ~~—~~create unique training experiences that may not be captured in broader studies. These challenges may directly influence students’ confidence and competence in splinting, raising questions about the effectiveness of current training models in adequately preparing them for clinical practice.” |
| - Include more international perspectives to situate your study within broader educational trends. | Refer to the literature review, revised discussions and limitations |
| **Literature Review**  - Expand the review to include international studies on occupational therapy students’ preparedness or clinical confidence. | Refer to revised literature review and strengths and limitations. |
| - Critically synthesize the literature to better support the need for this study. Consider models of experiential or competency-based education. | Refer to revised literature review and strengths and limitations |
| **Metodology** | N/A |
| Explain how saturation was determined and who conducted the data coding. Was intercoder agreement assessed? | Data saturation was achieved as the researchers collected sufficient data to draw the necessary conclusions, and collecting any further data did not produce value-added insights into the study.  Coding was conducted by two researchers and verified by the other two researchers to enhance reliability.  Intercoder agreement was assessed using a consensus , where discrepancies in coding were discussed and resolved collaboratively by all researchers to ensure consistency and rigour in theme development. |
| Clarify whether steps were taken to mitigate interviewer or analysis bias. | A structured question guide was developed to guide interviewers during interviews, additionally peer debriefing and member checking were used where possible to validate interpretations and ensure credibility of the findings. |
| Justify the sample size and explain participant variation in more detail. | The sample size was determined by data saturation, which was reached when no new themes or significant insights were emerging from the data.  The study included 16 undergraduate occupational therapy students from the University of KwaZulu-Natal (UKZN), comprising 5 males and 11 females. Of these, 6 participants were in their third year of study and 10 were in their fourth year. This variation in academic level allowed for a broader range of perspectives, reflecting both emerging and more advanced student experiences with splinting in hand rehabilitation.  The variation in gender was random. |
| **Results**  Present clearer distinctions between 3rd and 4th year responses if relevant to the themes. | Refer to “Theme three: Comparison of Readiness in 3rd Year and 4th Year” |
| Consider a visual table mapping quotes to subthemes to enhance clarity and transparency. | Refer to table 2 |
| **Discussion**  Engage with educational theory (e.g., Kolb’s Experiential Learning Cycle or Bandura’s self-efficacy theory) to better contextualize findings. | Included Kolb’s in the revised literature review and discussion |
| Discuss implications for curriculum design more deeply—how might findings inform changes in timing, delivery, or assessment of splinting training? | Refer to discussion under student- proposed solution for enhancing splinting training. |
| Expand discussion on the limitations, especially regarding generalizability beyond UKZN. | Refer to revised section on limitations and recommendations. “To improve the limitations of this research, future studies should consider flexible scheduling or virtual platforms to enable focus groups, which can enrich data depth...” |
| Reviewer #4: Review of article entitled: Undergraduate occupational therapy students’ perceptions of their preparedness for splinting in hand rehabilitation: an exploratory study at the University of KwaZulu-Natal- PONE-D-25-01929 | N/A |
| **General** |  |
| This worthwhile research identifies an area of occupational therapy (OT) undergraduate (UG) practical learning that is of importance but inadequately addressed in the institutional curriculum where the research was conducted. Reference is also made to the paucity of information in available literature on why students feel inadequately prepared for the use of splinting in clinical practice. Socio-economic barriers to the use of splinting are raised. The concern that poor splinting technique will deter graduates from specialising in hand rehabilitation is articulated. All these factors attest to the justification for the carrying out of this research. | Thank you |
| The use of qualitative research methodology is appropriate with attention being paid to trustworthiness measures, ethical considerations and thematic analysis. However, some more detail related to aspects of the data collection needs to be given. The collected data in the form of quotations is of good quality and rich in useful information which suggests the open-ended questions were well designed and delivered. | Refer to the revised methodology section |
| The interpretation of results and conclusions offer suggestions and solutions to mitigate the curricular problems with the current practical teaching of splinting. | Thank you |
| **Abstract:**  Well written, succinct and includes all the main points relevant to each section of the study. |  |
|  | Thank you |
| **Background**  Overall good organisation of information and relevant content.  Minor corrections | Thank you |
| Page 5, Line 67: Is the use of the word “occupational” necessary? | Removed |
| Page 5, line 68: As the wording occupational therapy is used a lot perhaps consider introducing the abbreviation OT here as in occupational therapy (OT). | Refer to manuscript under background OT abbreviation has been introduced. |
| Page 5, lines 77-78: This point needs a citation. | Citation has been added. |
| Page 5, lines 79-80: This sentence is a little confusing. Perhaps consider using the word modality.as in:” which serves as a modality that is a crucial therapeutic….” | A crucial aspect of hand rehabilitation is splinting, which serves as a modality that is a crucial therapeutic technique in this field. |
| Page 5, line 83: There are instances in the manuscript where references and wording are repeated as here. Please check for similar problems elsewhere in the document and correct them. | We revised the entire manuscript, identified and removed such. |
| Research question:  Minor corrections | N/A |
| Page 6, line 96: Suggest the addition of OT to students (to qualify who the students are) if using the abbreviation or occupational therapy students if writing in full. | Added: What are the perceptions of OT students on undergraduate splinting as a part of hand rehabilitation at the University of KwaZulu-Natal? |
| This aligns with terminology to describe students in the Aim and objectives. | Refer to manuscript terms align. |
| Aim:  Minor correction | N/A |
| Page 6, line 100: Could abbreviate occupational therapy to OT here. | Done: The study aimed to investigate the perceptions held by undergraduate OT students regarding splinting in hand rehabilitation at the University of KwaZulu-Natal. |
| **Literature review**  Minor corrections | N/A |
| Can apply the abbreviation OT where appropriate. | Refer to manuscript under literature review OT abbreviation used and throughout manuscript. |
| Page 7, lines 113-114: this sentence raises the questions- How will healthcare access be enhanced? Which population’s well-being will be improved? Please clarify. | Strengthening undergraduate occupational therapy training at UKZN particularly through practical experiences like splinting will help produce more competent graduates who are better prepared to serve in the public health sector. This can enhance access to rehabilitation services for underserved populations in KwaZulu-Natal and improve the well-being of individuals affected by hand injuries in these communities |
| Page 7, lines 131-133; This sentence is confusing. Do occupational therapists move to the private sector due to lack of resources like splinting materials? | More than 90% of OTs with over five years of experience in hand rehabilitation tend to move into the private sector due to barriers such as limited access to resources, physical environment, client follow up and high caseloads [8]. |
| Page 8, line135: I would query the use of “apparent “here because the rest of the sentence suggests a definite shortage of occupational therapists. | Revised: In 2018, a shortage of OTs was evident in KZN, where the division needed 19 OTs but had at most six positions filled across various public healthcare sectors. |
| Page 8, lines 138-140: “…the uneven distribution of occupational therapists is due to the limited number…” The addition of this wording helps with clarity. I would also suggest dividing the original long sentence into two starting the second one “ This makes it difficult…. | Revised to two sentences |
| Page 8, line148: humans | Changed to individuals |
| Page 8, line 151: Introduce the abbreviation multidisciplinary team (MDT) in this line as the abbreviation is used in line 153. | Multidisciplinary team(MDT) |
| Page 9, line 162: I am interested to know how psychology is involved in restoring the function of the hand… | “Hand rehabilitation aims to restore functional use by applying knowledge from physiology, anatomy, and psychology [4,14]. While physiology and anatomy guide the physical restoration of structures, psychology plays a critical role in addressing the emotional, cognitive, and behavioural factors that influence recovery. Following a hand injury, individuals may experience a wide range of emotional distress, including anxiety, depression, frustration, fear, and guilt [15]. The loss of hand function can also lead to feelings of insecurity, dependency, and perceived incompetence, further contributing to psychological strain [16]. Studies on work-related hand injuries have shown high incidences of stress-related conditions such as anxiety disorders, major depression, and post-traumatic stress disorder [17,18]. These psychological factors can negatively impact motivation, pain perception, coping ability, and ultimately, engagement and outcomes in therapy.” |
| Page 9, line 183: Abbreviation UKZN already introduced. | Corrected |
| Page 11, line 215: Abbreviate to MD. | Corrected |
| Page 11, line 218: “emphasises” | Corrected |
| **Methodology**  What was the initial question used in the semi-structured interview? Who conducted the interviews? Were different researchers involved? Were meetings held during data collection to make sure all researchers were following the same questioning technique and adapting to changes in questioning brought about from insights from the data collection process? Were any incentives offered to participants such as free data or vouchers etc? | The research team consisted of four student researchers under the supervision of an experienced academic. Each researcher was responsible for interviewing participants randomly selected from the consented pool. The same question guide was used by each researcher. No incentives. The interviews were conducted when the participants were on their residences and use the free Wi-Fi in their residences |
| Was an inductive method used to analyse data? | A hybrid approach was used during the thematic analysis, as thematic analysis allows for both inductive and deductive methods. While Braun and Clarke’s six-step process guided the analysis, some themes emerged inductively from the data, and others were informed deductively by existing literature and research objectives |
| Was verbatim transcription of interviews carried out timeously? Who did this? Was it done in Microsoft Word? | Verbatim transcription of all interviews was conducted using Microsoft Word Online by the respective student researchers who had conducted the interviews. While transcription was not done immediately after each interview, it was completed promptly after all interviews had been conducted, to ensure data consistency and quality. Each researcher transcribed their own interviews to maintain familiarity with the data and to confirm accuracy. |
| Who undertook the coding of the data? Was this done independently by researchers assigned to the task? | Data coding was done collaboratively by all student researchers, who independently coded the same transcripts initially. A consensus approach was used, where differences in interpretation were discussed during group meetings to ensure inter-coder reliability and methodological rigour. |
| Minor corrections  Page 12, line 243: “was followed…. were adhered to.” | “The COREQ 32-item checklist was followed, and ethical principles were adhered to.” |
| Page 12, line 257. Was approval for carrying out the research also obtained from the Head of the Occupational Therapy Department and the Dean of Student Affairs? This information could be added here or in the Ethical considerations section on Page 14. | Ethical clearance and gatekeeper permission were granted by the UKZN Biomedical Research Ethics Committee (BREC), ethics number BREC/007090/2024 |
| Pages 12 and 13, lines 259-261: How and where was this data stored? Was the data anonymised? Did the information sheet give the rights of participants to confidentiality and the right to stop participation at any time with no consequence? Perhaps add to line 267-268. | Added |
| Page 13, line 262: give reference for UKZN ethical policy. | Reference has been added |
| Page 13, line 277; I would suggest removing wording “refer to title section for their credentials.” | Removed |
| Page 13, line 281: Perhaps add anonymised data to negate the chance of the reader thinking that there is any chance that identifiers would allow participants to be identified. | Added |
| **Results**  Rich data were collected and this allowed for pertinent and comprehensive quotations to be realised. This section is well laid out and quotations are appropriately used. | Thank you |
| Page 16, Table 4.2; under Theme one, Subtheme 1.3, Theme three, Subtheme 3.2  I suggest that one of the quotes in the text under these Subthemes be moved from the text and used in the table. | 1.3: One text from subtheme 1,3 added to the table  3.2: No changed have been made |
| **Discussion**  Although there is no specified word limit for this journal, I would suggest that perhaps in some instances fewer quotations could be used under the themes/subthemes. Some quotations basically say the same thing so the most comprehensive could be chosen. The use of a 3rd year and a 4th year quotation is good where possible to highlight differences in perceptions on a theme by students in different years of study. | Results: No changes were made as more quotes from participants substantiate rigour to the findings.  Discussion: the entire section was revised |
| Page 31, line 648: It is not preferred to begin a sentence with an abbreviation… in this case UKZN. | Revised |
| In general, the main points relating to undergraduate occupational therapy students’ perceptions of the preparedness for splinting in hand rehabilitation are dealt with and discussed in the discussion. | N/A |
| Sometimes in qualitative research “outliers” are identified in the data collection. |  |
| Perhaps consider adding a short paragraph on the dangers associated with inadequate splint making instruction. | See revised discussion |
| For example: Could the lack of practice and competency in splint making and application lead to splints that are too “tight”? Could that lead to further problems for a patient? Or the fact that students recognised that the lack of adequate practice could lead to “endangering your client” and “it’s very dangerous to put a person in a splint they’re not prepared for.” Why? Does the available literature deal with this? |  |
| **Conclusion** | Thank you |
| Concise and relevant to the study findings. |  |
| **Limitations:** |  |
| Well-articulated and identifies the four points of limitation clearly. |  |
| **Recommendations:** |  |
| These are very useful suggestions for addressing the problems identified in the study. I hope that at least some of these well thought out and useful ideas will be implemented. |  |
| **Referencing:** |  |
| Sequence of numbering of citations starts being chronologically inconsistent on Page 12 after reference 17. I’m not sure citation 18 is included in the manuscript. Please check. | Revised. |

| Journal Requirements: | |
| --- | --- |
| 1.Please ensure that your manuscript meets PLOS ONE's style requirements, including those for file naming. The PLOS ONE style templates can be found at | Done |
| 2. We note that the grant information you provided in the ‘Funding Information’ and ‘Financial Disclosure’ sections do not match.  When you resubmit, please ensure that you provide the correct grant numbers for the awards you received for your study in the ‘Funding Information’ section. | Noted. Will do. |
| 3. In the online submission form, you indicated that “Requests for access to the data can be made by contacting the corresponding author.”.  All PLOS journals now require all data underlying the findings described in their manuscript to be freely available to other researchers, either 1. In a public repository, 2. Within the manuscript itself, or 3. Uploaded as supplementary information. | The data availability statement was updated as follows:  “All raw data files are available from Open Science Framework (OSF). The full reference is as follows: UKZN undergraduate research Raw Data. OSF; 2024. Available from: <https://osf.io/yvd7r>. Contact the corresponding author for further details. |
