# Supplementary material for: Undergraduate occupational therapy students’ perceptions of their preparedness for splinting in hand rehabilitation: An exploratory study at the University of KwaZulu-Natal
Source: PLoS One. 2026 Feb 13;21(2):e0343017. doi: 10.1371/journal.pone.0343017 (PMC12904425; doi:10.1371/journal.pone.0343017)
Supplement: Attachment — Submitted filename: Rebuttal letter Responses to academic editor and reviewers.docx [file pone.0343017.s003.docx]

**Rebuttal letter - responses to academic editor and reviewer(s)**

- Please refer to the main manuscript (with no track changes) for accuracy.

| **Reviewer's Responses to Questions** | **Authors’ comments** |
| --- | --- |
| Reviewer #3 | |
| However, occasional inconsistency in tense (e.g., shifting between present and past when describing methods or context). A language polish would ensure clarity and readiness for publication. | Refer to the manuscript regarding revised ‘language polish and occasional inconsistency in tense’. |
| The authors may wish to mention implications for global OT education, especially in low-resource or similar healthcare settings, to enhance relevance. | Refer to this addition under **strength**  “Moreover, although the study focused on a single institution in South Africa, the findings hold relevance for institutions worldwide. These results could be shared at international platforms such as the World Federation of Occupational Therapists conference, potentially sparking discussions on standardised approaches that would benefit training globally.” |
| Line 324: "Data saturation was achieved..." – consider briefly stating how. | Refer to the line that follow, i.e., “Data saturation was achieved when the researchers had collected sufficient data to draw the necessary conclusions, and further data collection yield no value-added insights into the study” |
| Reviewer #5 | |
| Lines 172-4 "traumatic hand injuries, accounting for 17.7% of all cases in KZN" and lines 668-90 "traumatic hand injuries, which constitute approximately 17.7% of cases in KZN" both misquote the source ref Naidoo, who actually states "In the province of KwaZulu-Natal (KZN) in South Africa (SA), trauma is extensive, constituting at least 17.8% of overall emergency cases", and who references the 17.8% statistic to a paper by Stewart that itself does not mention hand trauma. This wording is misleading. | Revised as follows:  Lines 172-4: Global trends indicate an increasing number of traumatic hand injuries [19], while just above 30% of traumatic injuries are hand-related in South Africa [20].  Lines 668-90: The above findings support and expand upon available global [19] and South African [20] research, which suggests a growing demand for therapists skilled in managing traumatic hand injuries. |
| Line 336. Please define reflexivity. | Sentence revised as follows:  Reflexivity, which entails critiquing one’s own beliefs and subjectivity and their influence on the research process on a continuous basis [27], was maintained throughout the study to minimise bias. This was supported by a transparent decision-making process and regular collaborative discussions with the research team. |
| The description of the participant selection process lacks sufficient detail. What was the population size from which the participants were selected? What were the specifics of the purposive sampling strategy? What measures did the authors take to avoid sampling bias from recruiting a nonrepresentative proportion of disgruntled students - for example, what wording was used to describe the purpose of the study to potential participants? | Refer to the following:  **Under population**   - The study was conducted at UKZN, Westville campus, and targeted a student cohort of just below 70 registered combined 3^rd^ and 4^th^ year Bachelor of Occupational Therapy students in the College of Health Sciences.   **Under Data collection process**   - A generic invite was sent to the 3^rd^ and 4^th^ year students per the following text “Class representatives of 3rd and 4th year OT students were contacted via WhatsApp and requested to distribute an information sheet along with a Google form. This form collected (prospective) participants' contact information, level of study, and sex, enabling the researchers to identify them and obtain informed consent. The information sheet outlined the study's purpose, explained that participation was entirely voluntary, and stated that participants could withdraw at any stage without consequence. It also detailed any potential benefits of participation and included contact information for the researchers, allowing participants to ask questions or seek further clarification in accordance with UKZN's ethics policy [20].” |
| Because of the repeated statements that inadequate time was devoted to hand splint instruction, please provide context to compare what proportion of a typical starting OT practice would be expected to involve hand splinting with what proportion of the current OT curriculum is devoted to hand splinting. | Refer to the second paragraph under “**Undergraduate occupational therapy training in hand rehabilitation**” that we inserted. The in-class hand lectures duration ranges from half a day (5 hours) to three full days (8 hours per day). |
| It might be assumed from the manuscript that the only instruction received on hand splinting was in person. What instructional reference materials were provided to students regarding indications and techniques of hand splinting? | Refer to the second paragraph under “**Undergraduate occupational therapy training in hand rehabilitation**” that we inserted.  Various tools and materials are used for hand related teaching, encompassing traditional lectures with slides, demonstration videos, recommended textbooks, and recent, contextually relevant published articles. |
| Reviewer #6 | |
| I think a re-work of the abstract to more clearly and simply explain the lack of preparedness that OT students feel through training is important. There are some aspects of the training that I think the authors assume the readers will know - for instance, how many years is this training supposed to be? It appears to be 4, but it is not clear to me. Different countries may have varying school lengths. Also, I am not clear on what "blocks" are, which are described in the abstract and in the paper itself. | Refer to the **second last** paragraph under “**Undergraduate occupational therapy training in hand rehabilitation**” that we inserted.  The four-year Occupational Therapy programme at UKZN structures its clinical fieldwork as ‘blocks’ – i.e., periods of supervised practice that begin in the second year and are progressively scaffolded through to the fourth year. These blocks expose students to various fields of occupational therapy, including occupational therapy applied to physical conditions. The overall structure is as follows:  • 2^nd^ year: Students attend clinical settings once a week for 8 weeks (no splint-making, as splint training begins in third year).  • 3^rd^ year: Students attend three times per week for 8 weeks and start fabricating splints.  • 4^th^ year: Students attend clinical settings daily for 6 weeks. |
| For more specifics, the formatting of the study responses (italics) in the paper helps delineate it from the rest of the paper, but making these sections isolated in table-form, may make reading the paper more easy. | No changes made. |

| **Journal Requirement** | |
| --- | --- |
| 1. If the reviewer comments include a recommendation to cite specific previously published works, please review and evaluate these publications to determine whether they are relevant and should be cited. There is no requirement to cite these works unless the editor has indicated otherwise. | Not applicable. |
| 2. Please review your reference list to ensure that it is complete and correct. If you have cited papers that have been retracted, please include the rationale for doing so in the manuscript text, or remove these references and replace them with relevant current references. Any changes to the reference list should be mentioned in the rebuttal letter that accompanies your revised manuscript. If you need to cite a retracted article, indicate the article’s retracted status in the References list and also include a citation and full reference for the retraction notice. | Done. No changes made. |
