# Supplementary material for: Undergraduate occupational therapy students’ perceptions of their preparedness for splinting in hand rehabilitation: An exploratory study at the University of KwaZulu-Natal
Source: PLoS One. 2026 Feb 13;21(2):e0343017. doi: 10.1371/journal.pone.0343017 (PMC12904425; doi:10.1371/journal.pone.0343017)
Supplement: Attachment — Submitted filename: Rebuttal_letter__Responses_to_academic_editor_and_reviewers_auresp_3.docx [file pone.0343017.s004.docx]

**Rebuttal letter - responses to academic editor and reviewer(s)**

- We have addressed all comments thoughtfully while maintaining brevity to ensure the manuscript remains precise.
- Please refer to the main manuscript (with no track changes) for accuracy.

| **Reviewer's Responses to Questions** | **Authors’ comments** |
| --- | --- |
| Reviewer #5 | |
| I have one remaining request. In my prior review, I stated: Lines 172-4 "traumatic hand injuries, accounting for 17.7% of all cases in KZN" and lines 668-90 "traumatic hand injuries, which constitute approximately 17.7% of cases in KZN" both misquote the source ref Naidoo, who actually states "In the province of KwaZuluNatal (KZN) in South Africa (SA), trauma is extensive, constituting at least 17.8% of overall emergency cases", and who references the 17.8% statistic to a paper by Stewart that itself does not mention hand trauma. This wording is misleading.  The authors responded:  Lines 172-4: Global trends indicate an increasing number of traumatic hand injuries [19], while just above 30% of traumatic injuries are hand-related in South Africa [20].  However, reference 19 (Stewart, 2017) does not mention trends in the number of hand injuries. Please either modify the wording or supply a reference which supports the statement of increasing numbers. | Revised to “Traumatic hand injuries remain a significant global health concern [19]”... |
| Reviewer #7 | |
| Reviewer #7: Major Comments  Experiential learning sequencing and confidence development in splinting education  This manuscript presents a technically sound qualitative exploration of undergraduate occupational therapy students’ perceptions of preparedness for splinting in hand rehabilitation. The study is methodologically appropriate, ethically conducted, and clearly reported. The results are well supported by participant quotations and align with existing literature regarding the perceived gap between theoretical instruction and practical confidence. The following comments are offered to strengthen interpretation and discussion rather than to suggest changes to study design or scope. | Thank you.  We have addressed all comments thoughtfully while maintaining brevity to ensure the manuscript remains precise. |
| Several findings indicate that students’ limited confidence in splinting may be influenced not only by the quantity of practical exposure, but also by how experiential learning opportunities are sequenced and reinforced over time. While participants frequently describe insufficient practice time or limited clinical exposure, the data suggest that learning opportunities may be episodic and unevenly structured, which may inhibit consolidation of procedural competence. | -Under discission, we added “This pattern seems to suggest that confidence is influenced by both exposure and its sequencing.”  -Under “Kolb’s Experiential Learning Theory” we revised the sentence as follows: “Kolb’s Experiential Learning Theory [7,23] contextualises this study’s findings, highlighting the limitations of curriculum structures that prioritise theory over practical training, which may hinder procedural skill consolidation.”  -Under discussion, under “Student-proposed solutions for enhancing splinting training”, see “The proposal for the earlier introduction of splinting aligns well with scaffolding learning principles [36], which advocate introducing foundational skills early and building on them systematically to foster competence and confidence. For instance, introducing basic splinting techniques and materials at a junior level could enhance students’ preparedness. By providing more time to practice and refine skills, this scaffolding approach could mitigate the confidence deficit observed in clinical practice.”  -The comment under “RECOMMENDATIONS AND IMPACT ON PRACTICE” is meant to address this comment “Students should be encouraged to pursue a hand rehabilitation clinical placement”. |
| The discussion could be strengthened by more explicitly situating the findings within a staged experiential learning process consistent with Kolb’s experiential learning theory, which the authors reference as a guiding framework. Participants appear to engage in reflective observation and abstract conceptualization through theoretical instruction, yet report limited opportunities for structured active experimentation across progressively complex contexts. This partial traversal of the experiential learning cycle may help explain why increased exposure alone does not consistently translate into confidence or perceived readiness. | Please refer to the revised discussion |
| Early stages of skill development may benefit from deliberate practice under low cognitive load, emphasizing repetition, immediate feedback, and error tolerance to support initial procedural encoding. As skills begin to consolidate, learners may require contextualized practice that introduces controlled human variability and tactile feedback, enabling translation from procedural familiarity to applied competence. Finally, learning experiences that approximate clinical complexity may support integration of technical execution with communication, situational awareness, and adaptive decision-making, thereby completing the experiential learning cycle. | See revised comment under “RECOMMENDATIONS AND IMPACT ON PRACTICE” starting that “Specifically, splinting should be introduced earlier in the curriculum, with more extended practice sessions in the classroom that build skills gradually, starting with basic tasks and advancing to complex practice to help students to develop competence progressively.” |
| Relatedly, several participants describe fluctuations or erosion of confidence between instructional or clinical blocks, suggesting that confidence development may be sensitive to continuity and reinforcement of practice. Episodic exposure without sufficient opportunities for consolidation may result in partial skill acquisition, leaving learners theoretically prepared but uncertain in execution. Framing confidence as a dynamic construct influenced by reinforcement and skill consolidation over time may provide a more nuanced interpretation of participant recommendations for increased practice time. | -Under discussion, under “Student readiness and preparedness”, see “Students’ confidence appeared to fluctuate between teaching and clinical periods, suggesting that irregular exposure may disrupt continuity in their learning. This highlights the value of practice that is revisited and reinforced over time rather than delivered in isolated blocks.”  -Under discission, under “Student-proposed solutions for enhancing splinting training”, see “These patterns indicate that confidence develops gradually and is strengthened through repeated reinforcement, offering a broader understanding of why students consistently request more opportunities to practise” |
| Incorporating these perspectives into the discussion would allow the authors to interpret calls for additional practice and curriculum expansion as a need for structured progression and longitudinal reinforcement, rather than solely increased exposure. This framing remains consistent with the qualitative findings and theoretical positioning of the study while strengthening its educational implications and transferability to other practice-oriented health professions programs. | Please refer to the comments above. |
| Minor Comments  1. Clarification of analytic approach  The methods section notes the use of thematic analysis guided by COREQ and references both inductive and deductive elements. A brief clarification of how deductive components were operationalized, and how inductive coding was protected from excessive theoretical constraint, may enhance transparency for readers less familiar with hybrid qualitative approaches. | Revised as follows “While Braun and Clarke’s six-step process, namely familiarising with the data, data coding, identifying themes, reviewing themes, defining and naming themes and producing the report [31], guided the analysis, some themes emerged inductively from the data, and others were informed deductively by existing literature and research objectives aligning with the study’s focus , e.g., ‘explore the OT students’ perceptions of their readiness and preparedness’. To safeguard the inductive component, initial codes were generated directly from participants’ words and independently reviewed by other researchers before being examined alongside deductive expectations, allowing unexpected insights to be retained.” |
| 2. Sample sufficiency and interpretive scope  The results are supported by rich participant quotations. Where appropriate, the authors may consider briefly clarifying whether the intent of the sample size was to achieve thematic saturation or depth of perspective, particularly given the exploratory nature of the study. | Revised “The researchers aimed for data saturation and sufficient depth of perspective to ensure meaningful understanding of participants’ experiences, which together strengthened the reliability of the study [28]” |
| 3. Alignment of recommendations and themes  The discussion may be further strengthened by explicitly linking participant-recommended solutions to specific themes, clarifying whether perceived gaps are interpreted primarily as curricular sequencing issues, clinical placement variability, or an interaction of both. | No changes made since the current recommendations are deemed sufficient |
| Reviewer #8 | |
| Dear Authors, thank you for your efforts in improving the presentation of the paper.  It is a valuable paper, as it delves into the issues surrounding competency-based training. | Noted with appreciation |
